# Efficacy and safety of Tuina therapy for children with combined allergic rhinitis and asthma syndrome in remission: a randomized controlled trial protocol

**DOI:** 10.3389/fmed.2026.1854540

**Published:** 2026-06-23

**Authors:** Xin Yun Chia, Junhao Cai, Dan Dai, Qing Ji, Shijian Liu, Wenyi Wang, Mengran Si, Yan Yang, Ping Lu, Xiaoqiu Wang, Sijie Dang, Yi Zhong, Shuxia Wang, Guimao Wang, Bin Xiao

**Affiliations:** 1 Shanghai Municipal Hospital of Traditional Chinese Medicine, Shanghai University of Traditional Chinese Medicine, Jing'an, Shanghai, China; 2 School of Acupuncture-Moxibustion and Tuina, Shanghai University of Traditional Chinese Medicine, Pudong, Shanghai, China; 3 Community Health Service Center of Nanhui New Town, Pudong, Shanghai, China

**Keywords:** allergic rhinitis (AR), asthma, combined allergic rhinitis and asthma syndrome (CARAS), pediatric tuina, traditional chinese medicine (TCM)

## Abstract

**Background:**

Combined allergic rhinitis and asthma syndrome (CARAS) is a prevalent chronic respiratory disorder among children, characterized by the coexistence of allergic rhinitis and asthma symptoms, thereby substantially impairing quality of life. Pediatric Tuina, a traditional Chinese manual therapy, has demonstrated potential in the management of pediatric respiratory conditions; however, its effectiveness in treating CARAS remains insufficiently studied.

**Objective:**

This trial aims to assess the clinical efficacy and safety of pediatric Tuina in the treatment of CARAS, with the objective of establishing an effective, safe, and convenient external therapy of traditional Chinese medicine for CARAS.

**Methods:**

A total of 92 children aged 6–14 years with CARAS in remission will be randomly assigned (1:1) to either the control group (conventional medication) or the intervention group (conventional medication plus pediatric Tuina). The intervention will be administered three times weekly for 4 weeks, followed by a 24-week follow-up. The primary outcome measure is the Pediatric Rhinoconjunctivitis Quality of Life Questionnaire (PRQLQ) score. Secondary outcomes include the Childhood Asthma Control Test (C-ACT) / Asthma Control Test (ACT) score and traditional Chinese medicine (TCM) symptom scores. Safety will be assessed by monitoring any adverse events during the treatment period.

**Discussion:**

This study will provide evidence for the efficacy and safety of pediatric Tuina in improving CARAS symptoms and quality of life in children.

**Clinical trial registration:**

https://itmctr.ccebtcm.org.cn/mgt/project/view/1966089236985806848, identifier [ITMCTR2025001712].

## 1 Introduction

### 1.1 Background

Combined allergic rhinitis and asthma syndrome (CARAS) is a syndrome characterized by concurrent allergic rhinitis (AR) and asthma, sharing standard pathophysiological mechanisms involving IgE-mediated hypersensitivity and immune dysregulation (1, 2). Allergic rhinitis and asthma are common inflammatory diseases of the airways that frequently coexist, a condition sometimes referred to as “united airway disease” (3). Epidemiological studies have consistently shown a strong association between the two conditions, with allergic rhinitis often preceding the development of asthma (4, 5). The prevalence of allergic rhinitis in patients with asthma is high, with estimates ranging from 80% to 90% (4). One study of 142 asthma patients in Tianjin, China, found that 68% also had allergic rhinitis (5). In a broader survey in Shenmu City, China, the prevalence of allergic rhinitis was 25.4%, and asthma was 9.4%, with 6.5% of the adult population having combined allergic rhinitis and asthma (6). Another study involving 650 asthma patients found that 85% had concomitant allergic rhinitis (7). A meta-analysis shows that in China, the comorbidity rate of AR and asthma in children is substantial, with 35.01% of AR children developing asthma and 54.93% of asthmatic children experiencing AR (8). Globally, among children, the estimated prevalence of asthma is 12.0% and of allergic rhinitis is 12.7% (9). CARAS in children leads to recurrent nasal symptoms (e.g., congestion, sneezing) and lower respiratory symptoms (e.g., wheezing, cough), affecting sleep, school performance, and long-term quality of life (10).

Current management of CARAS primarily relies on pharmacotherapy, such as montelukast and inhaled corticosteroids, which may be associated with adverse effects (e.g., growth suppression) and poor long-term adherence (11). Thus, safe and effective complementary therapies are urgently needed.

Pediatric Tuina, a non-pharmacological therapy rooted in Traditional Chinese Medicine (TCM), involves stimulating specific acupoints to regulate qi (vital energy) and blood circulation. It has been used to treat pediatric respiratory conditions, with studies showing efficacy in reducing symptoms of AR and asthma (12–17). For example, a randomized trial demonstrated that pediatric Tuina improved nasal symptoms in children with mild AR, with better long-term effects than loratadine (14). Another study reported that Tuina combined with medication reduced asthma exacerbations and improved lung function in children (17). However, no rigorous trials have specifically evaluated Tuina for CARAS. In summary, as a common chronic inflammatory airway disease in children, the current conventional treatment for CARAS is associated with long-term dependency and limitations. Pediatric Tuina massage, as a non-pharmacological adjuvant therapy, has shown promise for treating isolated allergic rhinitis or asthma. However, systematic research on its holistic efficacy, standardized protocols, and mechanisms of action for CARAS as a comorbid condition remains insufficient. This study aims to conduct a randomized controlled trial to test the following hypothesis: pediatric massage combined with conventional treatment is more effective than traditional treatment alone in controlling the comprehensive symptoms and improving the quality of life of children with CARAS. The findings of this study are expected to provide high-level evidence for a safe and effective non-pharmacological Traditional Chinese Medicine intervention in the clinical management of CARAS.

### 1.2 Objectives

Primary objective: to assess the clinical efficacy and safety of pediatric Tuina as an adjunctive therapy for children with CARAS in the remission phase.

Secondary objectives: to evaluate improvements in quality of life (PRQLQ), asthma control (C-ACT/ACT), TCM symptom scores, and safety profiles.

## 2 Methods

### 2.1 Study design

This is a single-center, randomized, superiority, parallel-group controlled trial with a 1:1 allocation ratio. This is a single-center trial. All participant recruitment, intervention, and assessment procedures will be conducted at the Shanghai Municipal Hospital of Traditional Chinese Medicine, Shanghai, China. The study will consist of a 4-week intervention phase and a 24-week follow-up phase. Outcome assessments shall be conducted at baseline (T0), following the intervention (T1, week 2; T2, week 4), and during follow-up periods (T3, week 8; T4, week 12; T5, week 28). Assessors will remain blinded to group allocation to mitigate potential bias.

Table 1 presents the study schedule.

**Table 1:** Timeline for the study protocol.

	Trial period						
	Enrollment		Post-randomization		Follow-up		Close-out
Timepoint	–T1 to T0	T0	T1	T2	T3	T4	T5
Enrollment	
Eligibility screen	X						
Informed consent	X						
Randomization		X					
Intervention or comparator							
Intervention group: conventional medication plus pediatric Tuina		X	X	X			
Comparator group: conventional medication		X	X	X			
Assessments							
Baseline		X	X	X	X	X	X
PRQLQ		X	X	X	X	X	X
C-ACT/ACT		X	X	X	X	X	X
TCM symptom scores		X	X	X	X	X	X
Safety evaluation		X	X	X			

PRQLQ, pediatric rhinoconjunctivitis quality of life questionnaire; C-ACT, childhood asthma control test; ACT, asthma control test.

### 2.2 Participants

#### 2.2.1 Inclusion criteria

a) Meeting the diagnostic criteria of Western medicine and TCM as specified in the “Expert Consensus on Integrated Traditional Chinese and Western Medicine Diagnosis and Treatment of Allergic Rhinitis-Asthma Syndrome” (10);
b) The disease is in remission stage;
c) Aged 6–14 years;
d) The child's guardian has formally signed the informed consent form.
e) Children aged 8 years and above have provided their informed consent by signing the appropriate form.

#### 2.2.2 Exclusion criteria

a) Patients experiencing nasal congestion and rhinorrhea attributable to acute rhinitis, vasomotor rhinitis, among other conditions.
b) Children with congenital nasal physiological abnormalities, such as nasal stenosis, deviated nasal septum, adenoid hypertrophy, and others.
c) Patients experiencing wheezing discomfort due to bacterial bronchitis, bronchial developmental malformations, airway foreign bodies, respiratory failure, bronchiectasis, chronic obstructive pulmonary disease, among other conditions.
d) Children with primary diseases such as cardiovascular, cerebrovascular, hepatic, renal, and hematopoietic system conditions.
e) Children participating in other research groups;
f) Individuals unable to cooperate with the treatment.
g) Ulcers, infections, or skin lesions at the acupoint application sites.

#### 2.2.3 Withdrawal criteria

1. Voluntary withdrawal by caregivers or participants.
2. Severe adverse events or exacerbation of CARAS necessitating emergency treatment.
3. Failure to comply with at least 30% of interventions or assessments.

### 2.3 Sample size calculation

The sample size was calculated based on the primary outcome (PRQLQ). Based on previous literature (18), we assumed a minimal clinically significant difference of 10 points in PRQLQ between groups to ensure a more conservative estimate of statistical power.

The sample size was estimated using the formula for comparing means between two independent samples:


$$\begin{array}{c} \text{n}=2\times {\left[\left(\text{Z}1-\text{α}{/}_{2}+\text{Z}1-\text{β}\right)\times \text{ σ}/\text{δ}\right]}^{2} \end{array}$$

Given:


$$\begin{array}{l} \text{ δ}=10,\text{σ}=8.21,\text{Z}1-\text{α}{/}_{2}=1.96\left(\text{α}=0.05\right),\\ \text{Z}1-\text{β}=1.28\left(\text{β}=\;0.1\right) \end{array}$$

Substituting these values yielded –*n* = 15 per group.

However, considering that the reference study used conventional treatment + Chinese herbal medicine + tuina therapy in the experimental group, while this study uses only conventional treatment + tuina (without herbal medicine), it was estimated that 60% of the effect in the reference study was attributable to herbal medicine and 40% to tuina. Therefore, the sample size was adjusted to^*^n = 15 ÷ 0.4 ≈ 38 per group.

Accounting for a potential dropout rate of ≤ 20%, the adjusted sample size was calculated as:


$$\begin{array}{c} {\text{n}}_{\text{adjusted}}=38\times 1.2\;\approx \;46\text{ per group}. \end{array}$$

The final sample size was set at 46 participants per group, for a total of 92, to ensure balanced baseline characteristics between groups.

### 2.4 Randomization and allocation concealment

Eligible participants will be randomized using a computer-generated random number sequence created with Python (version 3.10 or higher). The sequence with a 1:1 allocation ratio by an independent statistician who is not involved in participant recruitment or intervention. Allocation will be concealed using sequentially numbered, opaque envelopes. Investigators responsible for recruitment and intervention will be unblinded, while outcome assessors and data analysts will remain blinded.

**Figure 1:**
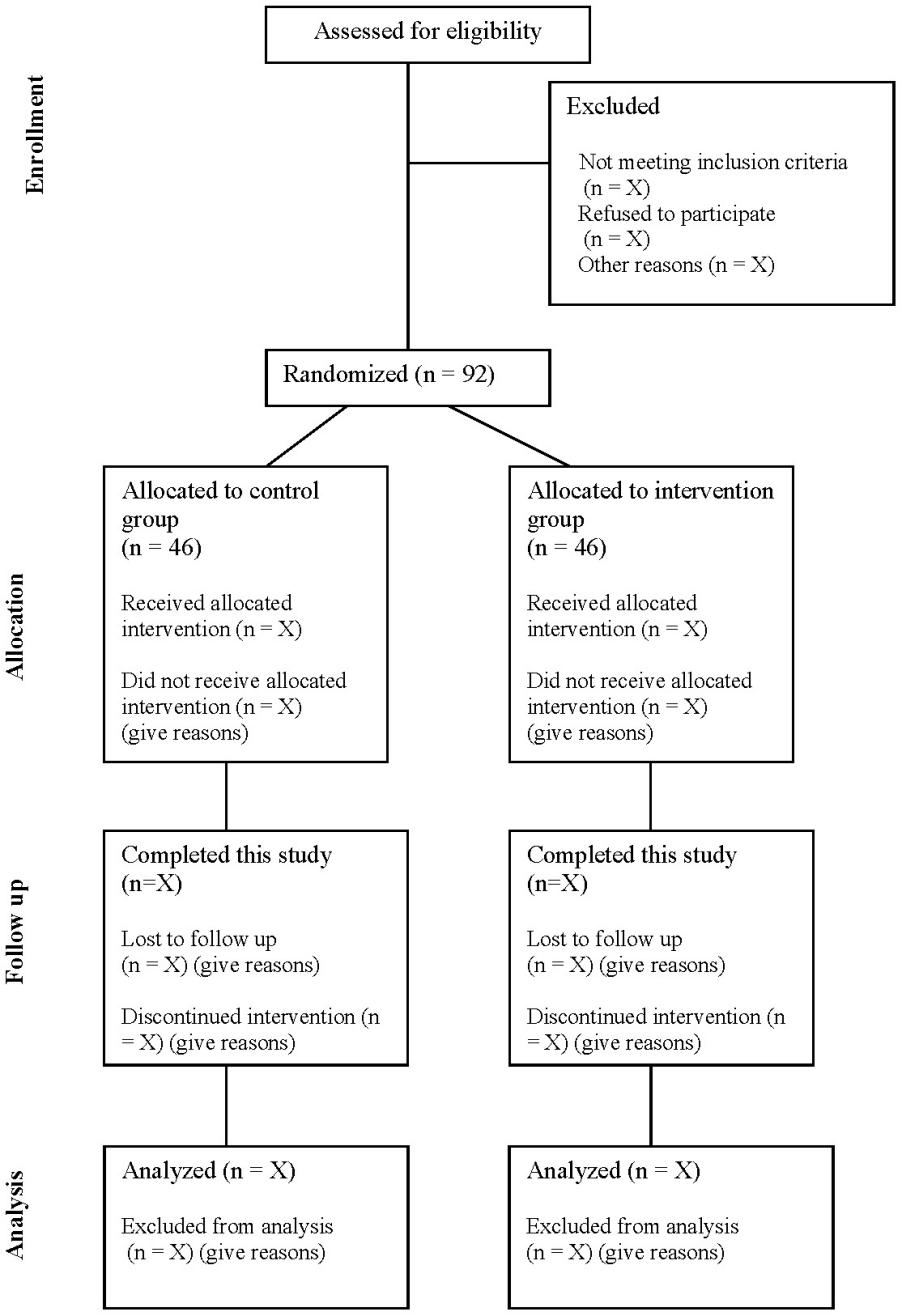
Study flow chart.

Figure 1 presents the research flow.

### 2.5 Interventions

#### 2.5.1 Control group: conventional medication

Participants will receive montelukast sodium chewable tablets: 5 mg once daily (bedtime).

Acute exacerbations during the trial will be managed with salbutamol aerosol or budesonide plus terbutaline nebulization, in accordance with clinical guidelines.

#### 2.5.2 Intervention group: conventional medication plus pediatric tuina

**Figure 2:**
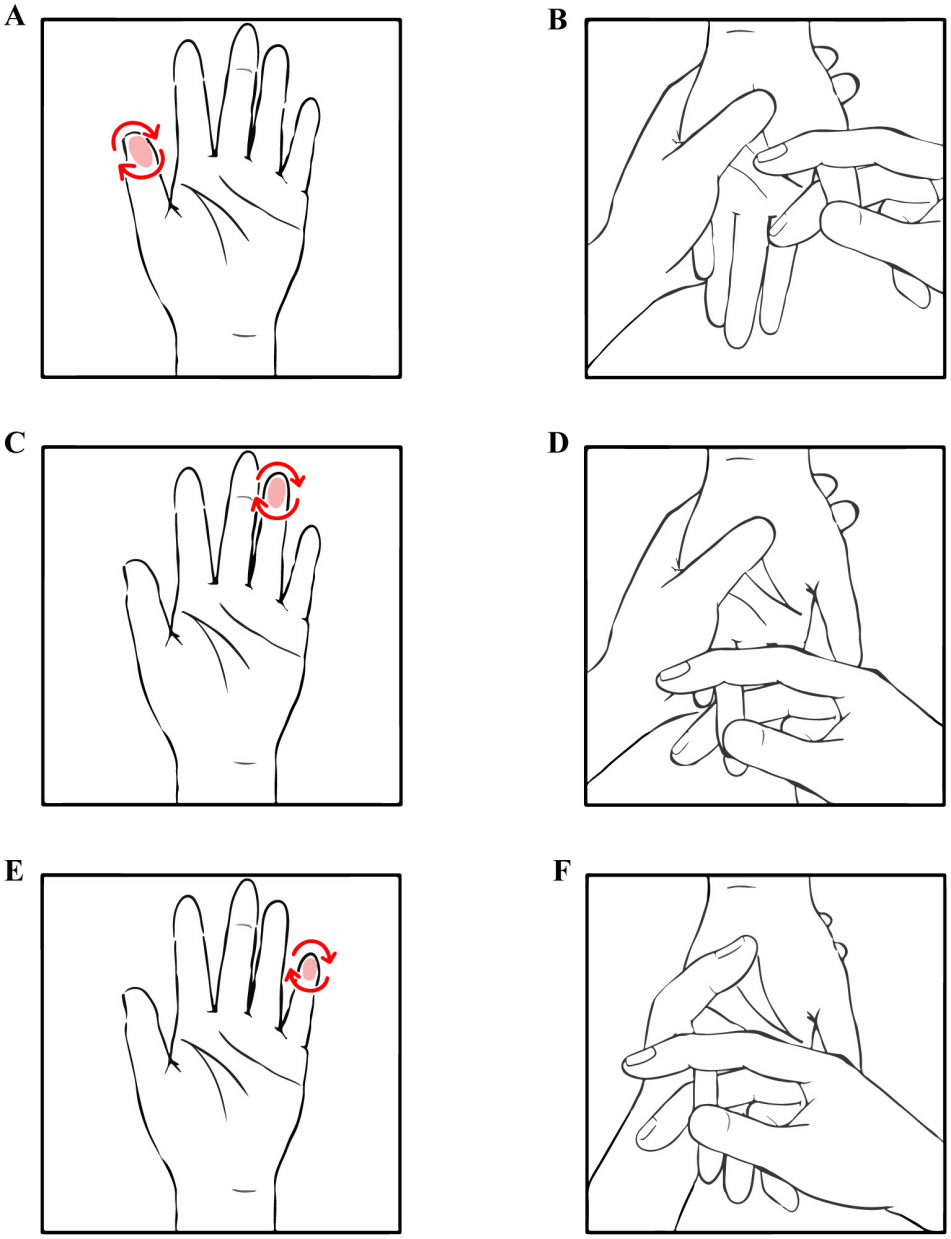
Spleen meridian, lung meridian, and kidney meridian. **(A)** spleen meridian, **(B)** pushing spleen meridian, **(C)** lung meridian. **(D)** pushing lung meridian, **(E)** kidney meridian, **(F)** pushing kidney meridian.

**Figure 3:**
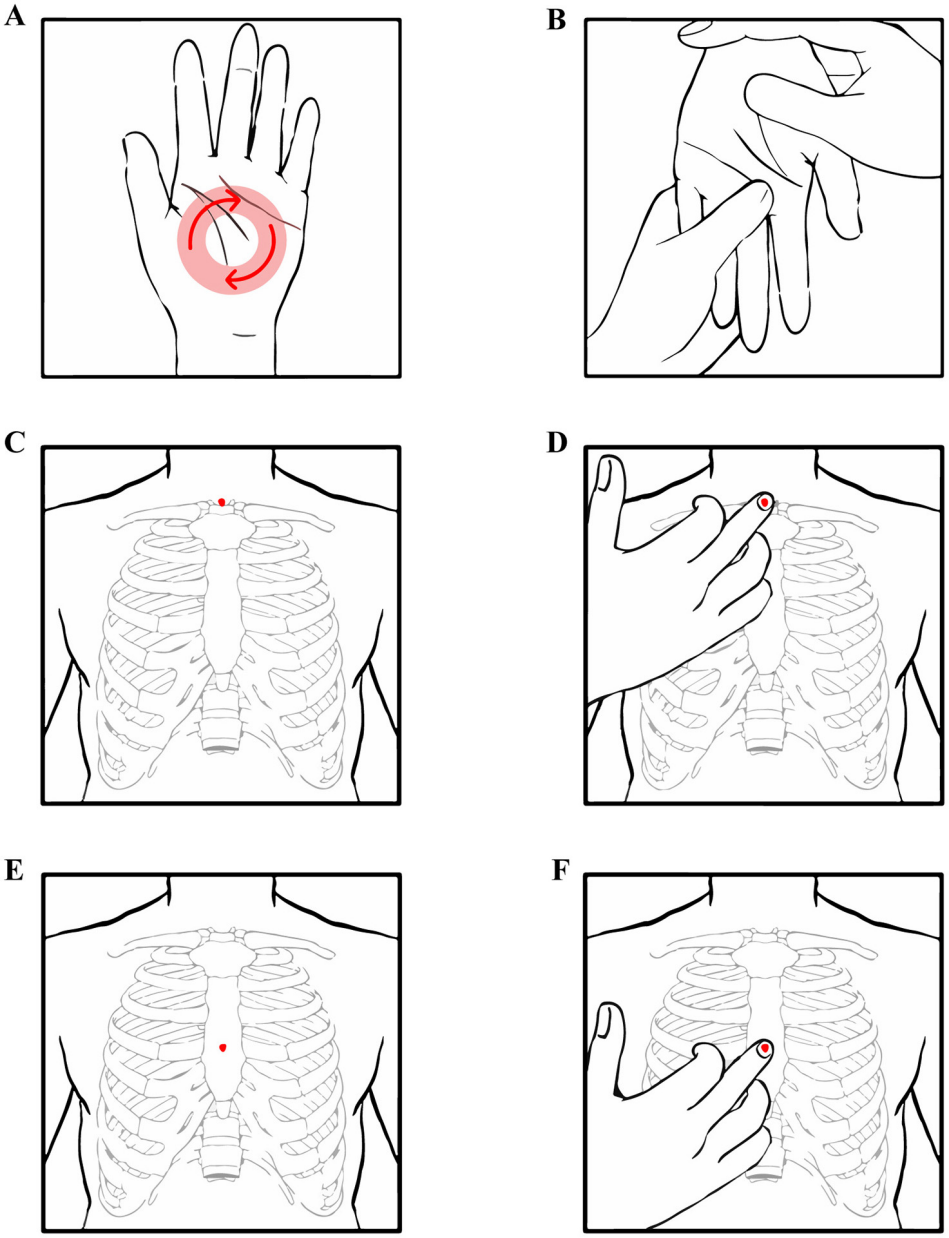
Inner eight trigrams, Tiantu and Danzhong. **(A)** Inner eight trigrams, **(B)** transporting inner eight trigrams, **(C)** Tiantu, **(D)** kneading Tiantu, **(E)** Danzhong, **(F)** kneading Danzhong.

**Figure 4:**
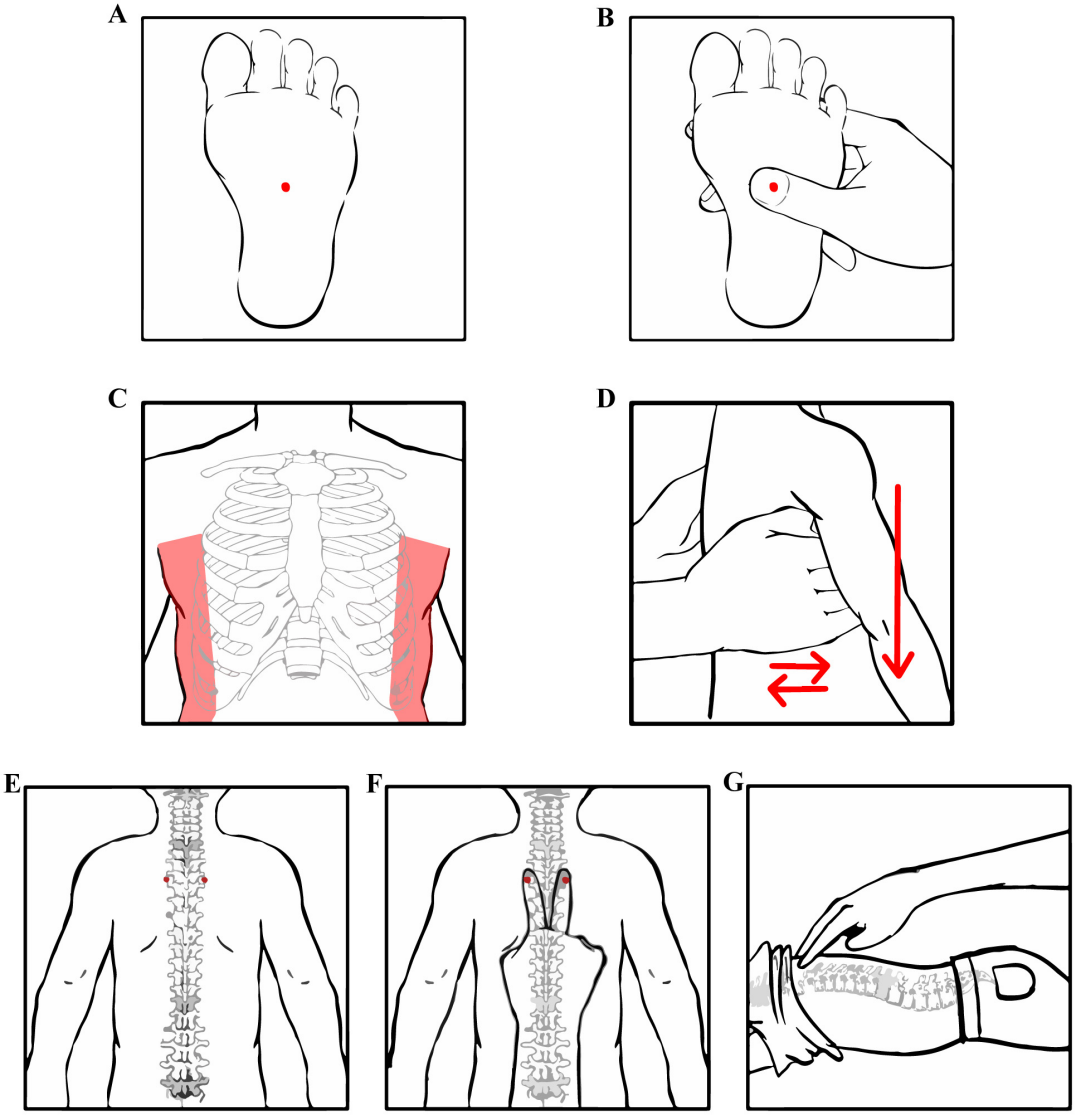
Yongquan, costovertebral region, and Feishu. **(A)** Yongquan, **(B)** kneading Yongquan, **(C)** costovertebral region, **(D)** twisting and rubbing costovertebral region, **(E)** Feishu, **(F)** kneading Feishu (back side), **(G)** kneading Feishu (lateral side).

**Figure 5:**
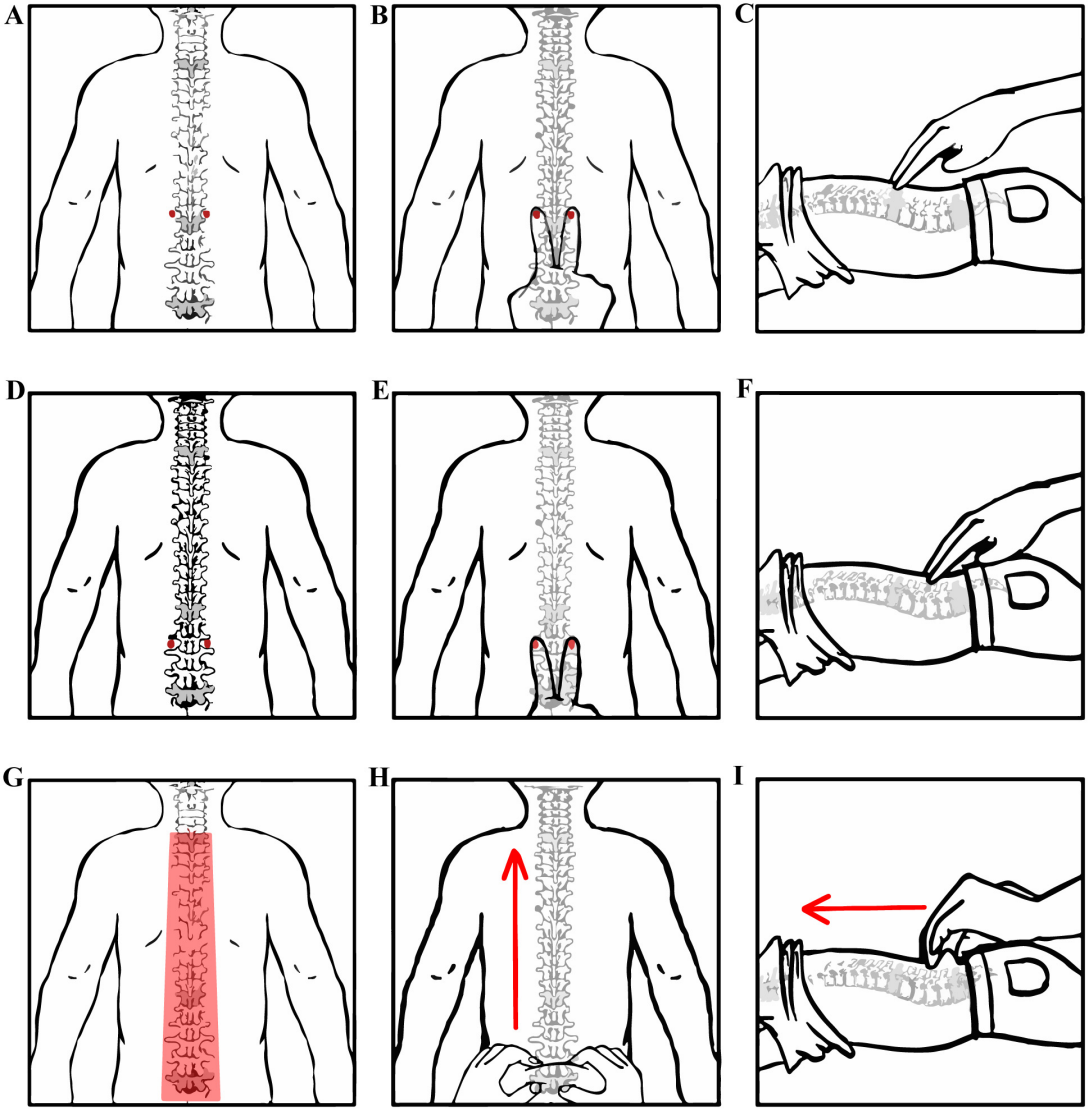
Pishu, Shenshu, and spine. **(A)** Pishu, **(B)** kneading Pishu (back side), **(C)** kneading Pishu (lateral side), **(D)** Shenshu, **(E)** kneading Shenshu (back side), **(F)** kneading Shenshu (lateral side), **(G)** spine (back side), **(H)** pinching spine, **(I)** pinching spine (lateral side).

Participants will receive the same medication as the control group, plus standardized pediatric Tuina. The Tuina protocol is based on TCM theory for “lung-spleen-kidney deficiency” (19) and includes:

Basic acupoints and techniques:

- Tonifying Spleen Meridian (300 rotations) [Figures 2A, B], Lung Meridian (300 rotations) [Figures 2C, D], Kidney Meridian (300 rotations) [Figures 2E, F].
- Circulating Inner Eight Trigrams (300 times) [Figures 3A, B].
- Kneading Tiantu (20 times) [Figures 3C, D], Danzhong (20 times) [Figures 3E, F], Yongquan (20 times) [Figures 4A, B].
- Rubbing the costovertebral region (50 times) [Figures 4C, D].
- Kneading Feishu (50 times) [Figures 4E–G], Pishu (50 times) [Figures 5A–C], Shenshu (50 times) [Figures 5D–F].
- Pinching the spine (5 times) [Figures 5G–I].

Additional movie files show this in more detail (see S1–S12 Movies).

Frequency and duration: 3 sessions/week for 4 weeks (total 12 sessions), each lasting 15–20 min. Tuina will be performed by certified therapists trained in the standardized protocol.

The anatomical localization of acupoints for pediatric Tuina strictly follows national standard as shown in Table 2 (20).

**Table 2:** Location of acupoints.

Acupoint	Location
Spleen meridian	The thumb ball, or the line from the tip to root on the radial border of thumb.
Lung meridian	The fingertip of the ring finger.
Kidney meridian	The fingertip of the little finger.
Inner eight trigrams	Taking palm center as the center, draw a circle with the radius from the center to the point at inner 2/3 of distance between palm center and transverse crease of middle finger root. Inner Eight Trigrams indicates the area within the circle.
Tiantu	In the depression, 0.6 cun superior to the midpoint on upper border of sternal notch, on conception vessel.
Danzhong	On the sternum, at the midpoint between two breasts.
Yongquan	On the sole of the foot, in the depression at the anterior part when the foot is curled. It is approximately located at the junction of the anterior 1/3 and posterior 2/3 of the line connecting the web margin tip between the 2nd and 3rd toes to the heel.
Costovertebral region	Hypochondriac regions from axillary line to ST 25 (tiān shu).
Feishu	1.5 cun lateral to lower border of the spinous process of the 3rd thoracic vertebra.
Pishu	1.5 cun lateral to lower border of the spinous process of the 11th thoracic vertebra.
Shenshu	1.5 cun lateral to lower border of the spinous process of the 2nd lumbar vertebra.
Spine	The straight line between DU 14 (dà zhui) and DU 1 (cháng qiáng).

### 2.6 Outcome measures

#### 2.6.1 Primary outcome

Quality of life: PRQLQ score (higher scores indicate poorer quality of life) at T0, T1, T2, T3, T4 and T5.

#### 2.6.2 Secondary outcomes

1. TCM symptom scores: assessing nasal (e.g., congestion, runny nose) and respiratory (e.g., cough, wheezing) symptoms, scored 0–3 (0 = absent, 3 = severe) (21).
2. Asthma control: C-ACT for 6–11 years old, ACT for 12–14 years old (higher scores indicate better control).

#### 2.6.3 Safety outcome

Adverse events (AEs) will be continuously monitored. Participants will be explicitly asked about any new or worsening symptoms at each treatment session and follow-up assessment. AEs specifically related to pediatric Tuina include, but are not limited to: intolerable pain, skin irritation, hematoma, bleeding, skin allergy, sweating, syncope, or dizziness. All AEs occurring during the trial will be assessed and documented in case report forms. Investigators will record detailed information on treatment measures, extent, duration, and the date of the AE. In the event of a serious AE, investigators must report it to the Ethics Committee of Shanghai Municipal Hospital of Traditional Chinese Medicine. The Ethics Committee will decide whether to withdraw a participant experiencing an AE, and will conduct audits every three months.

### 2.7 Data collection and management

Data will be recorded on case report forms (CRFs) and double-entered into an encrypted database. Missing data will be handled using multiple imputation. A Data Safety Monitoring Board will review data for safety and adherence to protocols. All electronic data will be stored using industry-standard encryption technology to prevent unauthorized access and data breaches. We will strictly control data access permissions to ensure that only authorized research team members can access this data, and passwords will be subject to regular audits to protect all access activities.

### 2.8 Quality control

Before trial initiation, all research personnel will receive comprehensive training on the study protocol, standardized assessment procedures, and documentation requirements. All measurement instruments will be regularly calibrated to ensure accuracy. To maintain consistency, the same group of outcome assessors will evaluate the same participants whenever possible throughout the study. Data quality will be ensured through multiple measures: data will be recorded on standardized case report forms by independent personnel, regular data audits will be performed, and the data management team will verify data authenticity and resolve any data quality issues.

### 2.9 Statistical analysis

All statistical analyses will be performed using Python (version 3.10 or higher). Data analysis will be conducted using standard scientific computing libraries, including NumPy, pandas, SciPy, and statsmodels. Data distribution will be assessed using the Shapiro–Wilk test. Normally distributed continuous variables will be presented as mean ± standard deviation (SD), while non-normally distributed data will be summarized as median with interquartile range (IQR). Categorical variables will be expressed as frequencies and percentages. A two-sided *p* value < 0.05 will be considered statistically significant.

All analyses will be conducted according to the intention-to-treat (ITT) principle, including all randomized participants in the groups to which they were originally assigned. A per-protocol (PP) analysis will also be performed including participants who complete the intervention without major protocol deviations. Safety analyses will include all participants who receive at least one session of the assigned intervention.

### 2.10 Ethics approval and consent to participate

This study will be conducted in accordance with the principles of the Declaration of Helsinki. The study protocol was approved by the Ethics Committee of [Shanghai Municipal Hospital of Traditional Chinese Medicine] (Approval No.: [2025SHL-KY-102-02]). Written informed consent will be obtained from caregivers and participants aged ≥8 years. Adverse events will be reported to the ethics committee within 24 h. Results will be published in peer-reviewed journals and presented at conferences.

## 3 Discussion

This study endeavors to assess the efficacy and safety of pediatric Tuina as an adjunctive therapy for CARAS in children through a randomized controlled trial. Should the trial outcomes substantiate the primary hypothesis—that the combined treatment cohort demonstrates significant enhancement over the conventional treatment group in terms of quality of life (PRQLQ score) and asthma management (C-ACT/ACT score)—the results will have substantial implications for clinical practice and research in integrative medicine.

First, at the clinical practice level, positive results would provide high-level evidence for the integrated management of CARAS. Pediatric Tuina, as a non-invasive, painless, and readily accepted external therapy, holds promise as an ideal “add-on” option. Without interfering with standard pharmacological treatment, it could synergistically improve coexisting upper- and lower-airway symptoms through TCM's holistic regulatory approach (such as ventilating the lungs, clearing the orifices, reinforcing healthy qi, and consolidating the root). This may reduce symptom fluctuations and enhance long-term treatment adherence and quality of life for affected children. Such an approach would be of significant value to families seeking alternative or complementary therapies to medication.

Secondly, at the level of academic research, this study aims to address a significant gap in the current field. Existing literature predominantly investigates interventions for allergic rhinitis or asthma separately; however, this research explicitly focuses on the syndromic entity of “one airway, one disease.” By using internationally recognized patient-reported outcome (PRO) measures and disease control tools, the findings will be positioned to align with mainstream international respiratory medicine research, thereby enhancing the comparability and credibility of TCM interventions within a contemporary medical framework. If the TCM symptom scores also show synchronous improvement, this would further support the advantage of individualized treatment plans based on syndrome differentiation in managing complex diseases.

Nevertheless, we acknowledge the limitations inherent in this study. The most prominent restriction concerns the intervention, which makes blinding of participants and operators exceedingly challenging, thereby potentially introducing performance bias and placebo effects. To partially address this issue, blinding will be implemented for outcome assessors and statisticians. Furthermore, because this study does not include biomarker detection, any observed clinical improvements will remain a “black box” regarding the underlying biological mechanisms (whether modulation of immune-inflammatory pathways is involved). Elucidation of these mechanisms will require future mechanistic exploratory trials. Additionally, although a 24-week follow-up is scheduled to evaluate mid- to long-term effects, the longevity of the intervention's benefits and its efficacy in preventing relapse in CARAS, a chronic condition, will require validation through studies with extended follow-up periods. Another limitation concerns the primary outcome measures. The PRQLQ only assesses rhinoconjunctivitis-related quality of life and does not capture the asthma component of CARAS, which constitutes a limitation of the present study. Future research should incorporate asthma-specific outcome measures, such as the Asthma Quality of Life Questionnaire (AQLQ) or lung function parameters, to enable a more comprehensive evaluation of CARAS.

In summary, this study is expected to provide key clinical effectiveness and safety data on pediatric Tuina for the treatment of CARAS. Should positive results be obtained, they would strongly promote the integration of this traditional Chinese therapy into modern comprehensive management guidelines for pediatric respiratory diseases and lay a solid foundation for subsequent research into mechanisms, protocol optimization, and health economic evaluation.
